# Comparing lesion and feature selections to predict progression in newly diagnosed DLBCL patients with FDG PET/CT radiomics features

**DOI:** 10.1007/s00259-022-05916-4

**Published:** 2022-08-04

**Authors:** Jakoba J. Eertink, Gerben J. C. Zwezerijnen, Matthijs C. F. Cysouw, Sanne E. Wiegers, Elisabeth A. G. Pfaehler, Pieternella J. Lugtenburg, Bronno van der Holt, Otto S. Hoekstra, Henrica C. W. de Vet, Josée M. Zijlstra, Ronald Boellaard

**Affiliations:** 1 Department of Hematology, Amsterdam UMC Location Vrije Universiteit Amsterdam, De Boelelaan 1117, 1081 HV Amsterdam, The Netherlands; 2 Cancer Center Amsterdam, Imaging and Biomarkers, Amsterdam, The Netherlands; 3 Radiology and Nuclear Medicine, Amsterdam UMC Location Vrije Universiteit Amsterdam, Amsterdam, The Netherlands; 4 Department of Nuclear Medicine, University Hospital Augsburg, Augsburg, Germany; 5 Department of Hematology, Erasmus MC Cancer Institute, University Medical Center Rotterdam, Wytemaweg 80, 3015 CN Rotterdam, the Netherlands; 6 Department of Hematology, HOVON Data Center, Erasmus MC Cancer Institute, Dr. Molewaterplein 40, 3015 GD Rotterdam, the Netherlands; 7 Epidemiology and Data Science, Amsterdam UMC Location Vrije Universiteit Amsterdam, Amsterdam, The Netherlands; 8 Amsterdam Public Health Research Institute, Methodology, Amsterdam, The Netherlands

**Keywords:** Diffuse-large-B-cell-lymphoma, Lesion selection, Radiomics, ^18^F-FDG-PET/CT, Prediction

## Abstract

**Purpose:**

Biomarkers that can accurately predict outcome in DLBCL patients are urgently needed. Radiomics features extracted from baseline [^18^F]-FDG PET/CT scans have shown promising results. This study aims to investigate which lesion- and feature-selection approaches/methods resulted in the best prediction of progression after 2 years.

**Methods:**

A total of 296 patients were included. 485 radiomics features (*n* = 5 conventional PET, *n* = 22 morphology, *n* = 50 intensity, *n* = 408 texture) were extracted for all individual lesions and at patient level, where all lesions were aggregated into one VOI. 18 features quantifying dissemination were extracted at patient level. Several lesion selection approaches were tested (largest or hottest lesion, patient level [all with/without dissemination], maximum or median of all lesions) and compared to the predictive value of our previously published model. Several data reduction methods were applied (principal component analysis, recursive feature elimination (RFE), factor analysis, and univariate selection). The predictive value of all models was tested using a fivefold cross-validation approach with 50 repeats with and without oversampling, yielding the mean cross-validated AUC (CV-AUC). Additionally, the relative importance of individual radiomics features was determined.

**Results:**

Models with conventional PET and dissemination features showed the highest predictive value (CV-AUC: 0.72–0.75). Dissemination features had the highest relative importance in these models. No lesion selection approach showed significantly higher predictive value compared to our previous model. Oversampling combined with RFE resulted in highest CV-AUCs.

**Conclusion:**

Regardless of the applied lesion selection or feature selection approach and feature reduction methods, patient level conventional PET features and dissemination features have the highest predictive value.

Trial registration number and date: EudraCT: 2006–005174-42, 01–08-2008.

**Supplementary Information:**

The online version contains supplementary material available at 10.1007/s00259-022-05916-4.

## Introduction

Diffuse large B-cell lymphoma (DLBCL) is the most common type of non-Hodgkin lymphoma and is associated with an aggressive disease course. Adding the monoclonal antibody rituximab to treatment regimens has improved outcome significantly [1–3]. However, still approximately 30% of patients with DLBCL experience disease progression or relapse, leading to poor outcome [4]. In rituximab-treated DLBCL patients, both the original International Prognostic Index (IPI) score [5] and other IPI variants such as the revised IPI and National Comprehensive Cancer Network IPI fail to identify a subgroup with a poor long-term survival (e.g., < 50%) [6], stressing the need to identify new biomarkers that can accurately select a specific subgroup with poor outcome when treated with standard chemo-immunotherapy.

Recent studies have shown that radiomics features extracted from baseline [^18^F]-fluorodeoxyglucose positron emission tomography computed tomography ([^18^F]FDG PET/CT) scans have promising predictive abilities in DLBCL [7–11]. Radiomics features provide detailed quantitative information regarding tumor morphology, texture, dissemination, and intensity, reflecting the tumor biology. In solid cancers, radiomics features are usually extracted from the primary lesion. However, radiomics analysis in lymphoma is more challenging due to the absence of one primary lesion in most patients and the often disseminated spread of the disease throughout the body in many different nodal and extranodal sites. Due to the high inter- and intra-tumor heterogeneity within patients, the metabolic tumor volume (MTV) at patient level best reflects disease burden. Therefore, some studies calculated radiomics features at patient level [7, 12]. As texture features become hard to interpret at patient level, other studies calculated radiomics features only for the lesion with the highest metabolic activity (highest maximum standardized uptake value (SUV_max_)) [8, 9], or for the lesion with the largest volume [10, 11].

We previously demonstrated that radiomics features at patient level are more predictive than radiomics features of the hottest and the largest lesion [12]. We now aimed to investigate how to aggregate information from multiple individual lesions in a patient to predict progression after 2 years, and whether this would improve prediction of progression after 2 years. We compared different lesion selection approaches and combined radiomics features from individual lesions with patient level radiomics features. Moreover, we explored the influence of different data reduction methods on model performance and investigated the feature importance of individual features in models.

## Methods

### Study population

DLBCL patients from the multicenter HOVON-84 trial (EudraCT: 2006–005,174-42) who had a baseline ^18^F-FDG PET/CT scan and had 2-year follow-up data available were included in this study. ^18^F-FDG PET/CT scans were included from 58 different hospitals*.* Because there were no differences in survival between randomization arms, all patients were included in this analysis [13]. Detailed inclusion and exclusion criteria of the HOVON-84 trial [13] and detailed quality control criteria for PET/CT imaging have been described elsewhere [12]. The study was approved by the Institutional Review Board and all participants gave written informed consent to participate.

### Quantitative image analysis

To match quality control criteria, the mean hepatic SUV should be between 1.3 and 3.0 and the plasma glucose less than 11 mmol/L [14]. When the hepatic SUV_mean_ was outside acceptable ranges, but the total image activity was between 50 and 80% of the total injected FDG activity, scans were still included. The majority of included scans was scanned according to EARL criteria [14]. Quantitative PET/CT analysis was performed using the ACCURATE tool [15]. Lesions were delineated using a fully automated preselection of ^18^F-FDG avid structures defined by an SUV ≥ 4.0 and a volume threshold of ≥ 3 mL [16]. Non-tumor regions were deleted and lymphoma lesions < 3 mL were added with single mouse clicks. Non-tumor ^18^F-FDG avid regions near tumor regions were manually removed. Delineations were performed under supervision of a nuclear medicine physician who was blinded to outcome.

### Feature extraction

Four hundred eighty features pertaining to morphology (*n* = 22), intensity (*n* = 50), and texture (*n* = 408) were extracted both for individual lesions and for the patient level volume of interest (VOI). Before feature calculation, all images were resampled to 2 × 2 × 2 mm voxel size using centered-grid tri-linear interpolation. In order to calculate textural features, the images were discretized with a fixed bin size of 0.25 SUV [17]. Texture features were based on the gray-level co-occurrence matrix, gray-level run length matrix, gray-level size zone matrix, gray-level distance zone matrix, neighborhood gray tone difference matrix, and neighboring gray-level dependence matrix with up to 8 matrix calculation methods. For the patient level VOI, all voxels belonging to the different lesions were processed if they were part of one VOI.

Furthermore, 18 additional dissemination features were extracted at patient level: the number of lesions, four features quantifying distance between lesions as suggested by Cottereau et al. [7], 10 features quantifying the differences in intensity between lesions, and three features quantifying the differences in volume between lesions. Additional information regarding the definitions of the dissemination features is presented in Supplemental Table 1 and Supplemental Fig. 1. Five conventional PET features were extracted from the original images without resampling: MTV, SUV_max_, SUV_peak_, SUV_mean_, and total lesion glycolysis. All image-processing and feature calculations were performed using RaCat software [18], which complies with the Image Biomarker Standardization Initiative (IBSI) standards [19].

**Fig. 1:**
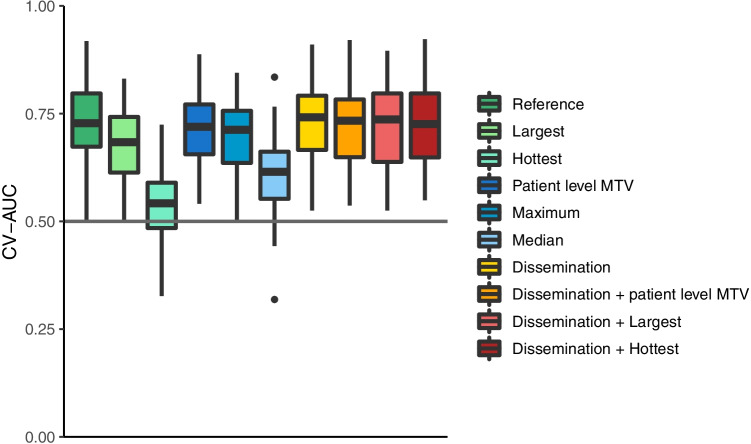
cross-validated AUCs for each prediction model using different lesion selection approaches

### Selected lesion and feature combinations

The predictive value of radiomics features of the following lesion and feature combinations was tested (hereafter referred to as lesion selection approaches):

1. MTV, SUV_peak_, and the maximum distance between the largest lesion and any other lesion at patient level (Dmax_bulk_) (reference) [12];
2. Largest lesion (largest, *n* = 480 radiomics features and *n* = 5 conventional PET features);
3. Hottest lesion (hottest, *n* = 480 radiomics features and *n* = 5 conventional PET features);
4. Radiomics features of all lesions summed into one VOI (patient-level MTV, *n* = 480 radiomics features and *n* = 5 conventional PET features);
5. Maximum value of radiomics features using all individual lesions per patient (maximum, *n* = 480 radiomics features and *n* = 5 conventional PET features);
6. Median value of radiomics features using all individual lesions per patient (median, *n* = 480 radiomics features and *n* = 5 conventional PET features);
7. Patient-level conventional PET features and dissemination features (dissemination, *n* = 18 radiomics features and *n* = 5 conventional PET features);
8. Patient-level conventional PET features and dissemination features and radiomics features of the patient level VOI model (“Dissemination + patient level MTV”, *n* = 498 radiomics features (480 + 18) and *n* = 5 conventional PET features);
9. Patient-level conventional PET features and dissemination features and radiomics features of largest lesion (“Dissemination + largest”, *n* = 498 radiomics features and *n* = 5 conventional PET features);
10. Patient-level conventional PET features and dissemination features and radiomics features of hottest lesion (“Dissemination + hottest”, *n* = 498 radiomics features and *n* = 5 conventional PET features).

As an explorative analysis, we tested the predictive value of radiomics features of the following lesion and feature combinations:

11. Patient-level conventional PET features and dissemination features and maximum value of radiomics features using all individual lesions per patient (“Dissemination + maximum”, *n* = 498 radiomics features and *n* = 5 conventional PET features);
12. Patient-level conventional PET features and dissemination features and median value of radiomics features using all individual lesions per patient (“Dissemination + median”, *n* = 498 radiomics features and *n* = 5 conventional PET features).

### Preprocessing methods

We tested the predictive value of the different models using logistic regression as classifier. Non-normally distributed features with a skewness in value distribution > 0.5 were log-transformed using the natural logarithm. Six different feature reduction methods were applied using the *Scikit-learn* library (version 0.24.1) in Python 3.6: (1) principal component analysis (PCA), where multiple orthogonal components are created to explain the maximum amount of variance in the data, (2) random forest (RF) recursive feature elimination (RFE), where feature selection is performed by iteratively training a model, ranking features, and then removing the lowest ranking features using a random forest algorithm, (3) RFE using a support vector machine algorithm, (4) RFE using a logistic regression algorithm, (5) univariate selection method based on ANOVA testing that retained the top 10 percentile features and (6) factor analysis (FA), where multiple components are created to explain variance in the data where components can model variance in every direction.

To correct for imbalance in patients experiencing progression and patients without progression after 2 years, oversampling of patients with progression was applied in each training set. Synthetic samples were generated with interpolated feature values using SMOTE [20], as implemented in the *skicit-learn* package in python. To assess the influence of preprocessing methods, models were also trained and cross-validated without feature reduction and oversampling.

### Statistical analysis

The binary endpoint of this study was progression after 2 years; follow-up started at the date of the baseline PET/CT scan. Patients who died or were lost to follow-up within 2 years were excluded. As a sensitivity analysis, we used progression-free survival after 2 years as binary endpoint for the optimal models with regards to oversampling and feature reduction.

To validate the prediction of progression after 2 years, a previously published approach was used [21]. In short, a fivefold cross-validation approach stratified for outcome was applied. In each fold, the model was trained on 80% of the data and validated on the unseen 20% of the data. To further limit chance findings, the cross-validation was repeated 50 times. To evaluate model performance, the area under the curve (AUC) was calculated from the receiver operating characteristics curve for each fold, yielding the cross-validated AUC (CV-AUC). To be able to compare the mean AUCs of the prediction models we used a framework that was proposed by van de Wiel et al. [22]. In each fold the AUCs of the two models were compared using the DeLong test [23]. The median of the *p* values of the different folds was reported as the final *p* value.

To assess feature importance, relative importance coefficients of each feature were derived using a random forest using the feature reduction method that showed the highest predictive value. The relative feature importance coefficients of all features in the model add up to one. PCA was excluded as feature reduction method as this does not yield interpretable features.

## Results

### Patients

Two hundred ninety-six patients were included in this analysis. The HOVON-84 patients originally included 574 patients. When the trial started, a baseline PET/CT scan was not mandatory. A total of 373 patients had a baseline PET/CT available, of which 317 adhered our quality control criteria. Fourteen patients died without signs of progression and seven patients were lost to follow up within 2 years. Fifty-two patients out of 296 showed progression within 2 years after baseline PET/CT. The majority of the included patients had advanced-stage disease, elevated lactate dehydrogenase levels, and high-intermediate or high risk IPI scores (Table 1). For 221 patients (75%), the largest lesion and hottest lesion were the same lesion. A mismatch between the hottest and largest lesion occurred most frequently when the volume of the largest lesion was less than 10 mL or, to a lesser extent, if the SUV_peak_ of the largest lesion was less than 10. In 73% (27 out of 37 patients) of the smaller lesions (< 10 mL), and in 39% (20 out of 52 patients) of the low-intensity lesions, a mismatch occurred, respectively. For the other mismatches, there was no clear relation between SUV_peak_, volume, lesions’ anatomical locations, or baseline clinical parameters. For 19 out of the 52 patients with progression (37%), there was no match between the hottest lesion and largest lesion.

**Table 1:** Patient characteristics of included patients

		*N* (%)
Age	Median (IQR)	65 (55–72)
	≤ 60 years	97 (33)
	> 60 years	199 (67)
Sex	Male	152 (51)
	Female	144 (49)
Ann Arbor stage	II	48 (16)
	III	62 (21)
	IV	186 (63)
LDH	normal	97 (33)
	> normal	199 (67)
Extranodal localisations	≤ 1	175 (59)
	> 1	121 (41)
WHO performance status	0	170 (57)
	1	87 (29)
	2	37 (13)
	missing	2 (1)
IPI	Low	48 (16)
	Low-intermediate	73 (25)
	High-intermediate	103 (35)
	High	72 (24)

Abbreviations: *LDH*, lactate dehydrogenase; *WHO*, World Health Organization; *IPI*, international prognostic index

### Comparison of lesion and feature selection approaches

Table 2 shows the optimal model parameters to predict progression after 2 years for each lesion or feature selection approach with regard to oversampling and feature reduction methods. The model that combined MTV, SUV_peak_, and Dmax_bulk_ (Model 1) yielded the highest CV-AUC (0.75 ± 0.09, Fig. 1). Patient-level dissemination features (Model 7; CV-AUC: 0.73 ± 0.09) predicted progression after 2 years better than lesional-based radiomics feature selection approaches (Models 2–6, all *p* > 0.05). Furthermore, adding dissemination features and patient-level conventional PET features resulted in higher CV-AUCs compared to the model performance of radiomics features of the largest (Model 2: CV-AUC: 0.72 ± 0.10 vs Model 9: 0.68 ± 0.08, *p* = 0.39) and the hottest lesion only (Model 3: CV-AUC: 0.68 ± 0.09 vs Model 10: 0.56 ± 0.10, *p* = 0.44). Moreover, adding dissemination features improved the predictive value of the patient level MTV model (Model 4: CV-AUC: 0.71 ± 0.08 vs Model 8: 0.70 ± 0.09, *p* = 0.437). Model performance was lowest using radiomics features of the hottest lesion (Model 3: CV-AUC 0.56 ± 0.10). Complex textural radiomics features did not have additional predictive abilities compared to dissemination features. There were no statistical differences between individual models due to high variance of model performance values. Results of the “maximum + dissemination” (Model 11) and “median + dissemination” (Model 12) models are presented in Supplemental Table 2. Model performances with progression free survival after 2 years as binary outcome are presented in Supplemental Table 3.

**Table 2:** Optimal model parameters to predict progression after 2 years for each lesion selection method

Model	Lesion selection approach	Oversampling	Feature selection	CV-AUC ± SD
1	Reference: MTV, SUVpeak, Dmaxbulk	Interpolate	None	0.75 ± 0.09
2	Largest	None	RFE-RF	0.68 ± 0.08
3	Hottest	Interpolate	FA	0.54 ± 0.09
4	Patient level MTV	Interpolate	FA	0.71 ± 0.08
5	Maximum	Interpolate	PCA	0.69 ± 0.10
6	Median	Interpolate	FA	0.61 ± 0.09
7	Dissemination	None	RFE-RF	0.73 ± 0.09
8	“Dissemination + patient level MTV”	Interpolate	None	0.72 ± 0.09
9	“Dissemination + largest”	Interpolate	RFE-RF	0.73 ± 0.09
10	“Dissemination + hottest”	Interpolate	None	0.72 ± 0.09

Abbreviations: *MTV*, metabolic tumor volume; *RF-RFE*, random forest recursive feature elimination; *FA*: factor analysis; *PCA*, principal component analysis

### Influence of preprocessing methods used

Oversampling resulted in higher CV-AUCs for all models except for the largest lesion (Model 2), where the CV-AUC after oversampling was lower (CV-AUC: 0.68 ± 0.08 vs 0.66 ± 0.08 after oversampling, Supplemental Table 2). For the dissemination feature model (Model 7), the CV-AUC was similar with or without oversampling (CV-AUC: 0.73 ± 0.09 for both models) when applying RF-RFE as feature reduction method. The most frequently selected data reduction methods were RF-RFE and FA. For all prediction models that included patient level dissemination and conventional features (Models 7–10), there was no difference in the CV-AUC when applying RF-RFE as feature reduction compared to no feature reduction. For the prediction models that used radiomics features from the hottest lesion (Model 3), patient level (Model 7), maximum averaging method (Model 5), and median averaging methods (Model 6), the CV-AUC decreased when applying RF-RFE as data reduction compared to the optimal data reduction method.

### Feature importance

**Fig. 2:**
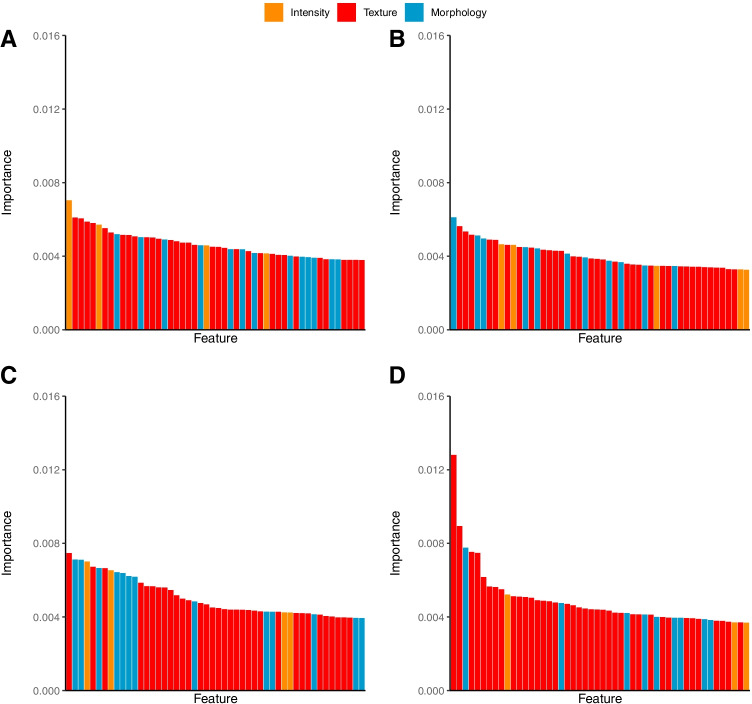
Feature importance of individual radiomics features with different feature selection approaches **A** largest, **B** hottest, **C** patient level MTV, and **D** maximum

**Fig. 3:**
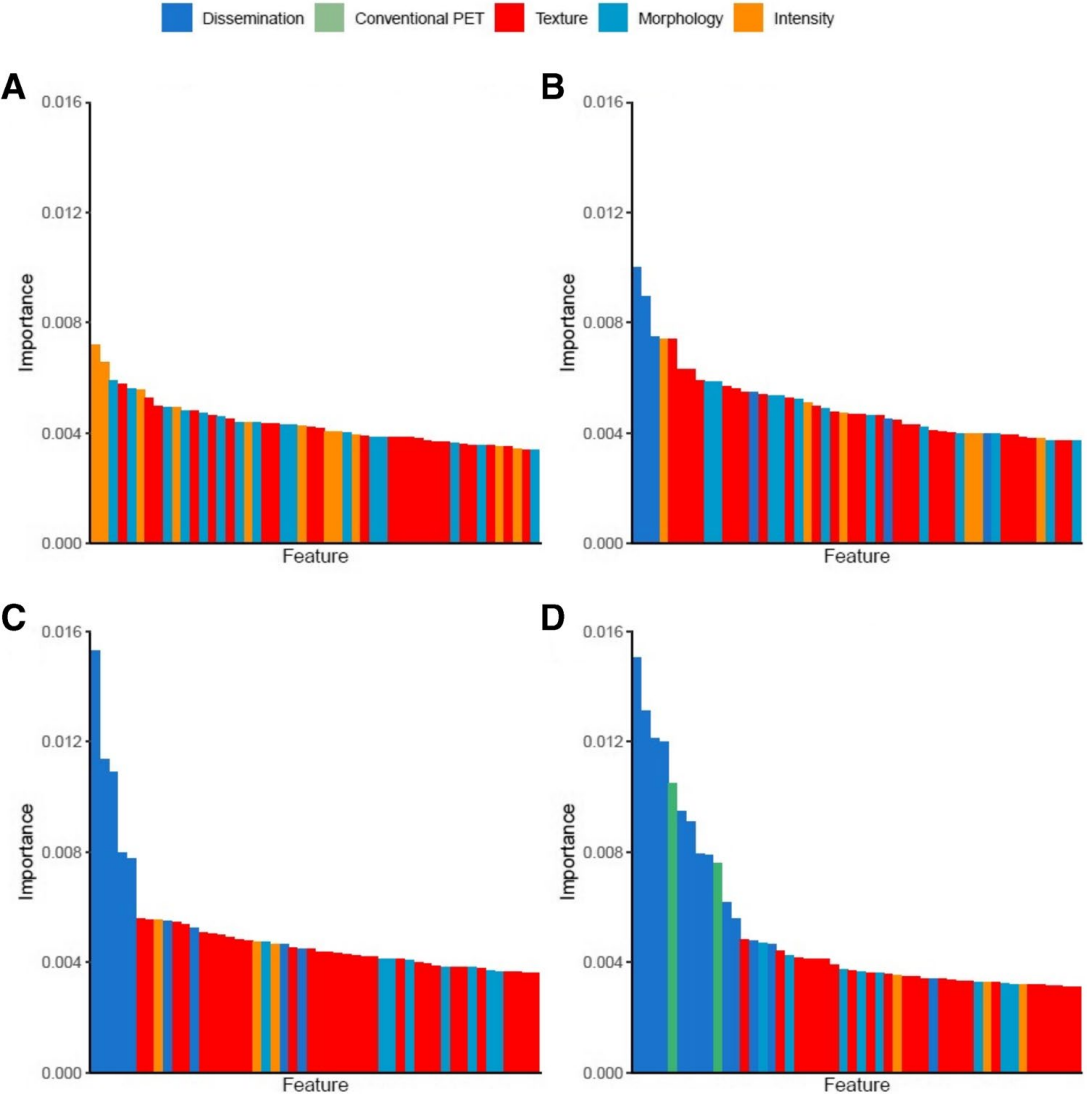
Feature importance of individual radiomics features with different feature selection approaches **A** Median, **B** “Dissemination + patient level MTV,” **C** “Dissemination + largest,” and **D** “Dissemination + hottest”

Feature importance per lesion or feature selection approach for each radiomics feature group is presented in Figs. 2 and 3. Dissemination features played an important role in predicting outcome. For all models that included dissemination features, the 3 most important features were always pertaining to dissemination. Dmax_bulk_ was the most important predictor in all these models. Dmax_bulk_correlated poorly with MTV (*r* = 0.20), also after correction for Ann Arbor stage. Three (Model 8), six (Model 9), seven (Model 7), and eight (Model 10) out of the 10 most important features were pertaining to dissemination, respectively. For all models without dissemination, the minimum intensity and either the morphological feature area density or the morphological feature volume density were in the top 10 most important features. Volume density approximate enclosing ellipsoid was included in the top 10% most important features for all models except for the “patient level + largest” model (Model 9). In all models, gray-level non-uniformity texture features and the morphological feature elongation were included in the top 10% of important features. In all models, except the maximum model, spherical disproportion and compactness were included. For all models, except the maximum and median models, the minimum histogram gradient was included in the 10% most important features. The most important features per model are presented in the Supplemental Tables 4, 5, 6, 7, 8, 9, 10, 11, 12, and 13.

## Discussion

This study showed that radiomics features at patient level had the highest predictive value. Prediction models based on more complex radiomics features with information of multiple lesions had no added predictive value compared to our previously published selection of more simple radiomics features when predicting progression after 2 years, regardless of the applied feature reduction method. Dissemination features showed high predictive abilities and improved outcome prediction for radiomics features extracted from the hottest lesion, largest lesion, or patient level MTV, although not significantly.

Historically speaking, the hottest lesions have been used to measure response during or after treatment [24, 25], and parameters quantifying uptake, such as SUV_max_ and SUV_peak_, have shown to be predictive in DLBCL [12, 26–28]. Therefore, it is surprising that radiomics features extracted from the hottest lesion have limited predictive value. For 75% of the patients in our dataset, the largest lesion also represented the hottest lesion. This was somewhat lower than the 84% reported in a recent study [10]. In our data, a mismatch occurred more frequently in smaller lesions. However, we could not find any clear correlation between PET parameters or clinical parameters, making it hard to hypothesize an explanation for this mismatch. We previously reported a lower CV-AUC of the hottest lesion, compared to the largest lesion and patient level radiomics features [12].

Currently, there is no valid approach to test whether the CV-AUCs of the various models are statistically significantly different, as there is no method to quantify correlation between trained models within cross-validation and there is an additional correlation between train-test data between CV iterations [29]. Therefore, we cannot definitely state which lesion selection approach has the highest predictive value. To be able to compare AUCs, we calculated the median AUC for each model for each fold. The disadvantage of this approach is that the *p* value is based on data of a single fold (20% of the data), resulting in low power to detect true differences. Therefore, the procedure was repeated 50 times with a random 20% sample of the data in order to obtain a reliable estimate of the *p* value using its median value over 50 repeats*.* Nevertheless, there were no significant differences between individual models, making them interchangeable. However, it seems that patient-level dissemination features play an important role. When including dissemination feature, the predictive value consistently increased for all models. A combination of MTV, SUV_peak_, and Dmax_bulk_ showed the best predictive abilities. Nonetheless, this CV-AUC is only marginally higher than the CV-AUC of other models that included dissemination features and patient-level conventional PET features. Yet, the model based on MTV, SUV_peak_, and Dmax_bulk_ might be preferred for translation into the clinic as these features are easy to understand and relate to disease characteristics that can be easily recognized in the PET image by eye.

There is a growing interest in radiomics features to predict outcome or select patients for innovative new treatment options, as more and more studies show their independent predictive value besides well-established clinical predictors [8, 10–12, 30, 31]. In order to implement radiomics in a clinical setting, user-friendliness is important. After extensively testing lesion and feature selection approaches combined with different data reduction methods, we could not find any added value for textural and morphological radiomics features. Moreover, textural features are known to have reproducibility and repeatability issues in a clinical setting [17], making feasibility of application of prediction models using textural features in clinical practice lower. Since the predictive value of dissemination features combined with conventional PET features was highest, it is advisable to calculate these features. Contrary to morphological and textural radiomics features, dissemination features are easy to interpret because they quantitatively reflect what can be visualized on PET/CT scans. They are also relatively simple to calculate and relatively insensitive to differences in acquisition, reconstruction, and delineation method [17, 32]. From an ease-of-use perspective, median and maximum lesion selection methods are more time-consuming and therefore not preferred since all individual lesions have to be processed individually to calculate radiomics features for each lesion. Moreover, the median prediction model showed limited discriminative power compared to other lesion selection methods. Radiomics features extracted from the patient level MTV (Model 4) are predictive of outcome. However, the interpretation of multi-cluster radiomics features is complex, both mathematically and clinically. Therefore, features extracted from this model might not be suitable for a clinical setting. Currently, there is no consensus on the optimal segmentation method in DLBCL, although the SUV4.0 method has been suggested [33]. However, we recently showed that the segmentation method does not influence the discriminative power of dissemination features [32].

Several other studies have evaluated the predictive value of baseline radiomics features in DLBCL. Aide et al. [11] showed that for the largest lesion, nine textural features (out of 19) were univariate significant [11]. Parvez et al. calculated 42 features for the 1–3 hottest lesions and reported that 3 textural features significantly predicted disease-free survival [9]. Decazes et al. showed that in a multivariate analysis IPI, chemotherapy, MTV, and the total volume surface ratio were all significant [31]. Two studies extracted the metabolic heterogeneity from the hottest lesion [8], or the largest lesion [10]. Both studies showed that patients with high MTV and high metabolic heterogeneity had significantly lower survival rates compared to patients with only one of these risk factors. Nonetheless, MTV was the only significant predictor of outcome in a multivariate analysis. Due to the different (numbers of) features that were extracted, it is hard to directly compare these studies. Generally speaking, our results confirm that radiomics features are predictive of outcome and have added value compared to MTV. Moreover, we extend on these findings by showing that dissemination features are very important and that adding complex textural radiomics features does not have additional predictive abilities compared to dissemination features.

Dissemination expressed as distance between lesions was first introduced by Cottereau et al., showing that dissemination was a predictor of outcome independent of MTV [7, 30]. In our study, Dmax_bulk_ consistently had the highest feature importance, indicating that dissemination is more important than MTV when predicting outcome. Our study adds to their findings by showing that dissemination features quantifying the differences in uptake or difference in volume between lesions also showed high predictive value.

By applying different lesion selection approaches on the same patient samples, we could directly compare their predictive value using progression after 2 years as outcome. Because our aim was to compare the predictive value of radiomics features using different lesion selection approaches, we did not add any clinical predictors. When developing a prediction model, adding clinical predictors to radiomics features showed improved prediction of outcome in DLBCL [12, 30] and other types of lymphoma [34, 35]. A limitation of this study was that we did not externally validate our findings in a separate cohort making our findings explorative, although we applied internal-validation by using cross-validation. More specifically, most patients who were included in this study had advanced stage disease; therefore, our results need to be validated in other cohorts with limited-stage DLBCL patients. Lastly, the majority of the patients that were included in this study did not experience progression, causing imbalance in outcome. We corrected for this by creating synthetic samples. CV-AUCs of datasets with and without synthetic samples were comparable for all models, yet an effect of class imbalance cannot be ruled out.

## Conclusion

Patient-level conventional PET features and dissemination features have the highest predictive value to predict progression after 2 years in DLBCL regardless of the applied lesion selection or feature selection approach and feature reduction methods. Textural and morphological radiomics features do not show additional predictive value compared to conventional PET and dissemination features. Moreover, these features are easy to understand and correspond to disease characteristics that can be easily recognized or seen in the PET image. Therefore, it is advised to extract dissemination features and conventional PET features from baseline PET/CT scans to optimally predict outcome.

## Supplementary Information

Below is the link to the electronic supplementary material.

Supplementary file1 (DOCX 309 KB)

## Data Availability

The datasets generated during and/or analyzed during the current study are available from the corresponding author on reasonable request.
