# Supplementary material for: Comparing lesion and feature selections to predict progression in newly diagnosed DLBCL patients with FDG PET/CT radiomics features
Source: Eur J Nucl Med Mol Imaging. 2022 Aug 4;49(13):4642–51. doi: 10.1007/s00259-022-05916-4 (PMC9606052; doi:10.1007/s00259-022-05916-4)
Supplement: Supplementary file 1 — Supplementary file1 (DOCX 309 KB) [file 259_2022_5916_MOESM1_ESM.docx]

**Supplementary information**

**Article title:** The optimal lesion and feature selection to predict progression in newly diagnosed DLBCL patients with FDG PET/CT radiomics features

**Journal name:** European Journal of Nuclear Medicine and Molecular Imaging

**Author names**: J.J. Eertink, G.J.C. Zwezerijnen, M.C.F. Cysouw, S.E. Wiegers, E.A.G. Pfaehler, P.J. Lugtenburg, O.S. Hoekstra, H.C.W. de Vet, R. Boellaard, J.M. Zijlstra

**Affiliation and e-mail address of the corresponding author:** Amsterdam UMC, Vrije Universiteit Amsterdam, department of Hematology, Cancer Center Amsterdam, De Boelelaan 1117, Amsterdam, Netherlands, e-mail: [j.eertink@amsterdamumc.nl](mailto:j.eertink@amsterdamumc.nl)

**Supplemental Table 1. Included dissemination features and their definition**

| **Name** | **Definition** |
| --- | --- |
| NumberLesions | Number of lesions |
| DmaxPatient | The maximum distance between 2 individual lesions |
| DmaxBulk | The maximum distance between the largest lesion and another lesion |
| SpreadPatient | The sum of the distances between all individual lesions |
| SpreadBulk | The sum of the distances between the largest lesion and all other lesions |
| VolSpreadBulk | The sum of differences in volume between the largest lesion and all other lesions |
| DvolPatient | The maximum difference in volume between 2 individual lesions |
| VolspreadPatient | The sum of differences in volume between all individual lesions |
| DSUVmaxBulk | The maximum difference in SUV_max_ between the largest lesion and any other lesion |
| DSUVmaxSumBulk | The sum of the differences in SUV_max_ between the largest lesion and all other lesions |
| DSUVmaxPatient | The maximum difference in SUV_max_ between 2 individual lesions |
| DSUVmaxSumPatient | The sum of the differences in SUV_max_ between all individual lesions |
| DSUVmaxSumHot | The sum of the differences in SUV_max_ between the lesion with the highest SUV_max_ and all other lesions |
| DSUVpeakBulk | The maximum difference in SUV_peak_ between the largest lesion and any other lesion |
| DSUVpeakSumBulk | The sum of the differences in SUV_peak_ between the largest lesion and all other lesions |
| DSUVpeakPatient | The maximum difference in SUV_peak_ between 2 individual lesions |
| DSUVpeakSumPatient | The sum of the differences in SUV_peak_ between all individual lesions |
| DSUVpeakSumHot | The sum of the differences in SUV_peak_ between the lesion with the highest SUV_peak_ and all other lesions |


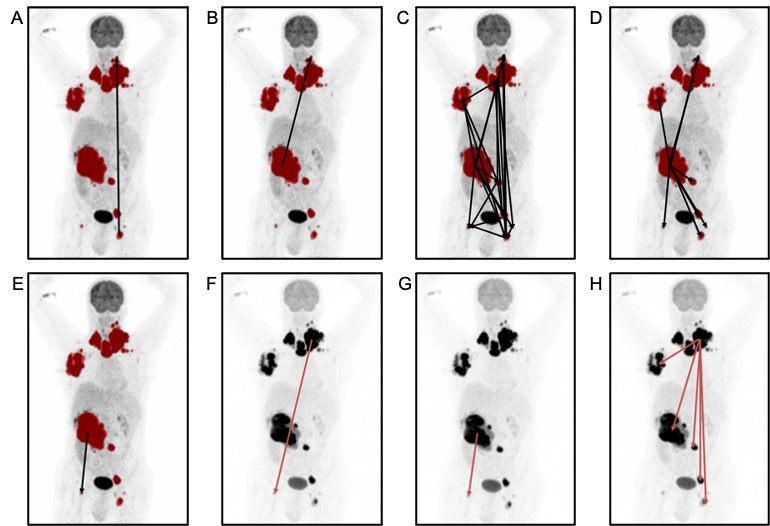


**Supplemental Figure 1. Visualisation of dissemination features**. A) DmaxPatient,

B) DmaxBulk, C) SpreadPatient, VolspreadPatient, DSUVmaxSumPatient,

DSUVpeakumPatient, D) SpreadBulk, VolSpreadBulk, E) DvolPatient, F) DSUVmaxPatient, DSUVpeakPatient, G) DSUVmaxBulk, DSUVpeakBulk, H)DSUVmaxSumHot, DSUVpeakSumHot,

**Supplemental Table 2. CV-AUCs with their standard deviation for all models and all feature selection options with and without oversampling.**

| **model** | **oversampling** | **feature selection** | **AUC mean** | **AUC std** |
| --- | --- | --- | --- | --- |
| Reference | None | None | 0.719 | 0.103 |
|  | Interpolate | None | 0.752 | 0.088 |
| Largest | None | None | 0.675 | 0.081 |
|  | None | PCA | 0.627 | 0.112 |
|  | None | RFE-RF | 0.676 | 0.082 |
|  | None | Univariate | 0.658 | 0.080 |
|  | None | PCA+RFE | 0.614 | 0.116 |
|  | None | FA | 0.547 | 0.073 |
|  | None | RFE-SVM | 0.631 | 0.089 |
|  | None | RFE-LR | 0.608 | 0.088 |
|  | Interpolate | None | 0.659 | 0.080 |
|  | Interpolate | PCA | 0.619 | 0.100 |
|  | Interpolate | RFE-RF | 0.658 | 0.076 |
|  | Interpolate | Univariate | 0.641 | 0.083 |
|  | Interpolate | PCA+RFE | 0.603 | 0.096 |
|  | Interpolate | FA | 0.639 | 0.089 |
|  | Interpolate | RFE-SVM | 0.625 | 0.082 |
|  | Interpolate | RFE-LR | 0.628 | 0.083 |
| Hottest | None | None | 0.490 | 0.035 |
|  | None | PCA | 0.536 | 0.079 |
|  | None | RFE-RF | 0.491 | 0.037 |
|  | None | Univariate | 0.490 | 0.035 |
|  | None | PCA+RFE | 0.515 | 0.074 |
|  | None | FA | 0.500 | 0.016 |
|  | None | RFE-SVM | 0.511 | 0.079 |
|  | None | RFE-LR | 0.496 | 0.035 |
|  | Interpolate | None | 0.511 | 0.073 |
|  | Interpolate | PCA | 0.525 | 0.079 |
|  | Interpolate | RFE-RF | 0.516 | 0.080 |
|  | Interpolate | Univariate | 0.520 | 0.081 |
|  | Interpolate | PCA+RFE | 0.502 | 0.078 |
|  | Interpolate | FA | 0.537 | 0.085 |
|  | Interpolate | RFE-SVM | 0.536 | 0.089 |
|  | Interpolate | RFE-LR | 0.501 | 0.093 |
| Patient level MTV | None | None | 0.693 | 0.072 |
|  | None | PCA | 0.635 | 0.128 |
|  | None | RFE-RF | 0.690 | 0.084 |
|  | None | Univariate | 0.699 | 0.083 |
|  | None | PCA+RFE | 0.621 | 0.125 |
|  | None | FA | 0.650 | 0.093 |
|  | None | RFE-SVM | 0.663 | 0.080 |
|  | None | RFE-LR | 0.528 | 0.056 |
|  | Interpolate | None | 0.704 | 0.081 |
|  | Interpolate | PCA | 0.640 | 0.130 |
|  | Interpolate | RFE-RF | 0.701 | 0.090 |
|  | Interpolate | Univariate | 0.695 | 0.091 |
|  | Interpolate | PCA+RFE | 0.620 | 0.124 |
|  | Interpolate | FA | 0.711 | 0.085 |
|  | Interpolate | RFE-SVM | 0.648 | 0.085 |
|  | Interpolate | RFE-LR | 0.641 | 0.079 |
| Maximum | None | None | 0.659 | 0.088 |
|  | None | PCA | 0.687 | 0.098 |
|  | None | RFE-RF | 0.660 | 0.087 |
|  | None | Univariate | 0.631 | 0.080 |
|  | None | PCA+RFE | 0.685 | 0.092 |
|  | None | FA | 0.681 | 0.072 |
|  | None | RFE-SVM | 0.598 | 0.086 |
|  | None | RFE-LR | 0.644 | 0.081 |
|  | Interpolate | None | 0.644 | 0.088 |
|  | Interpolate | PCA | 0.689 | 0.095 |
|  | Interpolate | RFE-RF | 0.644 | 0.086 |
|  | Interpolate | Univariate | 0.620 | 0.069 |
|  | Interpolate | PCA+RFE | 0.680 | 0.087 |
|  | Interpolate | FA | 0.684 | 0.072 |
|  | Interpolate | RFE-SVM | 0.598 | 0.091 |
|  | Interpolate | RFE-LR | 0.652 | 0.073 |
| Median | None | None | 0.493 | 0.026 |
|  | None | PCA | 0.521 | 0.083 |
|  | None | RFE-RF | 0.493 | 0.026 |
|  | None | Univariate | 0.498 | 0.035 |
|  | None | PCA+RFE | 0.514 | 0.085 |
|  | None | FA | 0.529 | 0.071 |
|  | None | RFE-SVM | 0.507 | 0.078 |
|  | None | RFE-LR | 0.527 | 0.069 |
|  | Interpolate | None | 0.529 | 0.094 |
|  | Interpolate | PCA | 0.508 | 0.081 |
|  | Interpolate | RFE-RF | 0.528 | 0.093 |
|  | Interpolate | Univariate | 0.532 | 0.095 |
|  | Interpolate | PCA+RFE | 0.515 | 0.085 |
|  | Interpolate | FA | 0.607 | 0.092 |
|  | Interpolate | RFE-SVM | 0.502 | 0.092 |
|  | Interpolate | RFE-LR | 0.533 | 0.084 |
| Dissemination | None | None | 0.730 | 0.093 |
|  | None | PCA | 0.720 | 0.091 |
|  | None | RFE-RF | 0.732 | 0.090 |
|  | None | Univariate | 0.662 | 0.087 |
|  | None | PCA+RFE | 0.708 | 0.095 |
|  | None | FA | 0.696 | 0.090 |
|  | None | RFE-SVM | 0.730 | 0.093 |
|  | None | RFE-LR | 0.728 | 0.092 |
|  | Interpolate | None | 0.725 | 0.095 |
|  | Interpolate | PCA | 0.731 | 0.096 |
|  | Interpolate | RFE-RF | 0.729 | 0.092 |
|  | Interpolate | Univariate | 0.677 | 0.100 |
|  | Interpolate | PCA+RFE | 0.718 | 0.102 |
|  | Interpolate | FA | 0.720 | 0.081 |
|  | Interpolate | RFE-SVM | 0.726 | 0.095 |
|  | Interpolate | RFE-LR | 0.726 | 0.092 |
| ‘Dissemination + Patient level MTV’ | None | None | 0.693 | 0.080 |
|  | None | PCA | 0.610 | 0.131 |
|  | None | RFE-RF | 0.693 | 0.079 |
|  | None | Univariate | 0.700 | 0.086 |
|  | None | PCA+RFE | 0.625 | 0.117 |
|  | None | FA | 0.648 | 0.087 |
|  | None | RFE-SVM | 0.636 | 0.095 |
|  | None | RFE-LR | 0.552 | 0.092 |
|  | Interpolate | None | 0.723 | 0.088 |
|  | Interpolate | PCA | 0.611 | 0.134 |
|  | Interpolate | RFE-RF | 0.722 | 0.089 |
|  | Interpolate | Univariate | 0.707 | 0.096 |
|  | Interpolate | PCA+RFE | 0.630 | 0.120 |
|  | Interpolate | FA | 0.704 | 0.087 |
|  | Interpolate | RFE-SVM | 0.635 | 0.093 |
|  | Interpolate | RFE-LR | 0.674 | 0.078 |
| ‘Dissemination + largest’ | None | None | 0.701 | 0.089 |
|  | None | PCA | 0.598 | 0.130 |
|  | None | RFE-RF | 0.706 | 0.084 |
|  | None | Univariate | 0.681 | 0.085 |
|  | None | PCA+RFE | 0.610 | 0.122 |
|  | None | FA | 0.557 | 0.073 |
|  | None | RFE-SVM | 0.626 | 0.084 |
|  | None | RFE-LR | 0.656 | 0.126 |
|  | Interpolate | None | 0.721 | 0.100 |
|  | Interpolate | PCA | 0.607 | 0.119 |
|  | Interpolate | RFE-RF | 0.726 | 0.094 |
|  | Interpolate | Univariate | 0.669 | 0.089 |
|  | Interpolate | PCA+RFE | 0.618 | 0.120 |
|  | Interpolate | FA | 0.660 | 0.088 |
|  | Interpolate | RFE-SVM | 0.631 | 0.094 |
|  | Interpolate | RFE-LR | 0.701 | 0.090 |
| ‘Dissemination + hottest’ | None | None | 0.651 | 0.084 |
|  | None | PCA | 0.580 | 0.090 |
|  | None | RFE-RF | 0.651 | 0.084 |
|  | None | Univariate | 0.662 | 0.078 |
|  | None | PCA+RFE | 0.579 | 0.090 |
|  | None | FA | 0.502 | 0.026 |
|  | None | RFE-SVM | 0.598 | 0.095 |
|  | None | RFE-LR | 0.499 | 0.030 |
|  | Interpolate | None | 0.724 | 0.090 |
|  | Interpolate | PCA | 0.593 | 0.103 |
|  | Interpolate | RFE-RF | 0.721 | 0.094 |
|  | Interpolate | Univariate | 0.713 | 0.095 |
|  | Interpolate | PCA+RFE | 0.606 | 0.102 |
|  | Interpolate | FA | 0.584 | 0.105 |
|  | Interpolate | RFE-SVM | 0.649 | 0.093 |
| ‘Dissemination + maximum’ | None | None | 0.683 | 0.071 |
|  | None | PCA | 0.654 | 0.119 |
|  | None | RFE-RF | 0.688 | 0.063 |
|  | None | Univariate | 0.692 | 0.072 |
|  | None | PCA+RFE | 0.618 | 0.121 |
|  | None | FA | 0.679 | 0.081 |
|  | None | RFE-SVM | 0.636 | 0.081 |
|  | None | RFE-LR | 0.593 | 0.104 |
|  | Interpolate | None | 0.704 | 0.080 |
|  | Interpolate | PCA | 0.661 | 0.122 |
|  | Interpolate | RFE-RF | 0.706 | 0.075 |
|  | Interpolate | Univariate | 0.676 | 0.086 |
|  | Interpolate | PCA+RFE | 0.609 | 0.126 |
|  | Interpolate | FA | 0.690 | 0.074 |
|  | Interpolate | RFE-SVM | 0.634 | 0.086 |
|  | Interpolate | RFE-LR | 0.653 | 0.073 |
| ‘Dissemination + median’ | None | None | 0.674 | 0.081 |
|  | None | PCA | 0.583 | 0.087 |
|  | None | RFE-RF | 0.668 | 0.084 |
|  | None | Univariate | 0.683 | 0.074 |
|  | None | PCA+RFE | 0.591 | 0.921 |
|  | None | FA | 0.627 | 0.102 |
|  | None | RFE-SVM | 0.650 | 0.092 |
|  | None | RFE-LR | 0.514 | 0.047 |
|  | Interpolate | None | 0.677 | 0.077 |
|  | Interpolate | PCA | 0.607 | 0.101 |
|  | Interpolate | RFE-RF | 0.675 | 0.083 |
|  | Interpolate | Univariate | 0.676 | 0.075 |
|  | Interpolate | PCA+RFE | 0.578 | 0.091 |
|  | Interpolate | FA | 0.696 | 0.091 |
|  | Interpolate | RFE-SVM | 0.649 | 0.091 |
|  | Interpolate | RFE-LR | 0.507 | 0.081 |

**Supplemental Table 3. Model performances using optimal model parameters using progression after 2 years and progression and death after 2 years as outcome**

| Model | Lesion selection approach | CV-AUC ± SD  Progression | CV-AUC ± SD  Progression & death |
| --- | --- | --- | --- |
| 1 | MTV, SUVpeak, Dmaxbulk | 0.75 ± 0.09 | 0.68 ± 0.07 |
| 2 | Largest | 0.68 ± 0.08 | 0.63 ± 0.07 |
| 3 | Hottest | 0.54 ± 0.09 | 0.52 ± 0.08 |
| 4 | Patient level MTV | 0.71 ± 0.08 | 0.69 ± 0.06 |
| 5 | Maximum | 0.69 ± 0.10 | 0.63 ± 0.10 |
| 6 | Median | 0.61 ± 0.09 | 0.57 ± 0.09 |
| 7 | Dissemination | 0.73 ± 0.09 | 0.60 ± 0.08 |
| 8 | ‘Dissemination + patient level MTV’ | 0.72 ± 0.09 | 0.68 ± 0.07 |
| 9 | ‘Dissemination + largest’ | 0.73 ± 0.09 | 0.67 ± 0.07 |
| 10 | ‘Dissemination + hottest’ | 0.72 ± 0.09 | 0.67 ± 0.06 |

**Supplemental Table 4. Feature importances of the Reference model (model 1)**

| **Feature** | **Feature group** | **Feature importance** |
| --- | --- | --- |
| DmaxBulk | Dissemination | 0.35016 |
| ExactVolume | First order | 0.34129 |
| global intensity peak | First order | 0.30854 |

**Supplemental Table 5. Feature importances of the most important features of the Largest model (model 2)**

| **Feature** | **Feature group** | **Feature importance** |
| --- | --- | --- |
| Minimum histogram gradient | Intensity Histogram | 0.00704 |
| Low dependence low grey level emphasis | Texture NGLDM 3D | 0.00610 |
| inverse difference moment normalised | Texture GLCM 2D | 0.00605 |
| busyness | Texture NGTDM 2D | 0.00587 |
| Grey level variance | Texture GLDZM 2D | 0.00581 |
| minimum | Statistics | 0.00571 |
| inverse difference normalised | Texture GLCM 3D | 0.00552 |
| Grey level non uniformity | Texture GLDZM 2D | 0.00529 |
| area density AABB | Morphology | 0.00520 |
| strength | Texture NGDTM 3D | 0.00516 |
| correlation | Texture GLCM 2D | 0.00515 |
| Grey level non uniformity | Texture GLSZM 2D | 0.00508 |
| elongation | Morphology | 0.00504 |
| Grey level non uniformity | Texture GLRLM 2D | 0.00503 |
| busyness | Texture NGTDM 3D | 0.00501 |
| correlation | Texture GLCM 2D | 0.00495 |
| Gearys C | Morphology | 0.00490 |
| Grey level non uniformity | Texture NGLDM 2D | 0.00488 |
| Small distance high grey level emphasis | Texture GLDZM 3D | 0.00481 |
| correlation | Texture GLCM 3D | 0.00474 |
| inverse difference moment normalised | Texture GLCM 3D | 0.00473 |
| inverse difference moment normalised | Texture GLCM 2D | 0.00461 |
| Compactness 1 | Morphology | 0.00459 |
| volume at intensity fraction | Intensity volume | 0.00458 |
| Large zone emphasis | Texture GLSZM 2D | 0.00451 |
| Low dependence low grey level emphasis | Texture NGLDM 2D | 0.00450 |
| Large zone high grey level emphasis | Texture GLSZM 2D | 0.00445 |
| center of mass shift | Morphology | 0.00439 |
| coarseness | Texture NGTDM 2D | 0.00439 |
| Compactness 2 | Morphology | 0.00438 |
| dependence Count Energy | Texture NGLDM 2D | 0.00427 |
| maximum 3D diameter | Morphology | 0.00418 |
| Large distance low grey level emphasis | Texture GLDZM 2 | 0.00417 |
| Maximum histogram gradient | Intensity Histogram | 0.00415 |
| Low dependence low grey level emphasis | Texture NGLDM 2D | 0.00413 |
| Grey level non uniformity | Texture GLRLM 2D | 0.00407 |
| inverse difference moment normalised | Texture GLCM 3D | 0.00406 |
| sphericity | Morphology | 0.00402 |
| High dependence high grey level emphasis | Texture NGLDM 2D | 0.00398 |
| least axis length | Morphology | 0.00397 |
| Spherical disproportion | Morphology | 0.00395 |
| volume density AEE | Morphology | 0.00392 |
| coarseness | Texture NGTDM 3D | 0.00390 |
| High dependence high grey level emphasis | Texture NGLDM 3D | 0.00383 |
| minor axis length | Morphology | 0.00383 |
| asphericity | Morphology | 0.00383 |
| Large distance low grey level emphasis | Texture GLDZM 2D | 0.00380 |
| Zone size variance | Texture GLSZM 2D | 0.00380 |
| Large distance low grey level emphasis | Texture GLDZM 3D | 0.00380 |
| first measure of information correlation | Texture GLCM 2D | 0.00379 |

**Supplemental Table 6. Feature importances of the most important features of the Hottest model (model 3)**

| **Feature** | **Feature group** | **Feature importance** |
| --- | --- | --- |
| busyness | Texture | 0.00639 |
| area density AABB | Morphology | 0.00596 |
| Low dependence low grey level emphasis | Texture NGLDM 3D | 0.00534 |
| joint maximum | Texture GLCM 2D | 0.00534 |
| Low dependence low grey level emphasis | Texture NGLDM 2D | 0.00530 |
| vol density AEE | Morphology | 0.00479 |
| dependence Count Energy | Texture NGLDM 2D | 0.00460 |
| angular second moment | Texture GLCM 2D | 0.00458 |
| Large distance low grey level emphasis | Texture GLDZM 2D | 0.00454 |
| volume at int fraction 90 | intensity volume | 0.00442 |
| Gearys C | Morphology | 0.00439 |
| elongation | Morphology | 0.00438 |
| asphericity | Morphology | 0.00435 |
| Large zone low grey level emphasis | Texture GLSZM 2D | 0.00432 |
| Minimum histogram gradient | Intensity histogram | 0.00427 |
| High dependence low grey level emphasis | Texture NGLDM 2D | 0.00424 |
| minimum | Statistics | 0.00423 |
| Large zone low grey level emphasis | Texture GLSZM 3D | 0.00423 |
| strength | Texture NGTDM 3D | 0.00413 |
| Minimum histogram gradient grey level | Intensity histogram | 0.00410 |
| inverse difference normalised | Texture GLCM 3D | 0.00409 |
| volume at int fraction 10 | intensity volume | 0.00399 |
| Grey level variance | Texture GLDZM 2D | 0.00398 |
| sphericity | Morphology | 0.00396 |
| inverse difference moment normalised | Texture GLCM 2D | 0.00395 |
| flatness | Morphology | 0.00390 |
| Compactness 2 | Morphology | 0.00380 |
| vol density AABB | Morphology | 0.00379 |
| busyness | Texture NGTDM 2D | 0.00368 |
| Large zone high grey level emphasis | Texture GLSZM 3D | 0.00368 |
| joint maximum | Texture GLCM 2D | 0.00365 |
| Grey level non uniformity | Texture NGLDM 2D | 0.00364 |
| Grey level non uniformity normalized | Texture GLRLM 2D | 0.00363 |
| inverse difference normalised | Texture GLCM 2D | 0.00358 |
| Small distance high grey level emphasis | Texture GLDZM 3D | 0.00352 |
| second measure of information correlation | Texture GLCM 2D | 0.00350 |
| cluster shade | Texture GLCM 2D | 0.00349 |
| first measure of information correlation | Texture GLCM 2D | 0.00345 |
| busyness | Texture NGTDM 3D | 0.00344 |
| first measure of information correlation | Texture GLCM 2D | 0.00344 |
| Grey level non uniformity | Texture GLDZM 2D | 0.00342 |
| Spherical disproportion | Morphology | 0.00342 |
| Low dependence low grey level emphasis | Texture NGLDM 2D | 0.00341 |
| first measure of information correlation | Texture GLCM 3D | 0.00339 |
| Grey level non uniformity | Texture GLDZM 2D | 0.00338 |
| Morans I | Morphology | 0.00336 |
| High dependence high grey level emphasis | Texture NGLDM 3D | 0.00335 |
| Zone size non uniformity normalized | Texture GLSZM 2D | 0.00331 |
| strength | Texture NGDTM 2D | 0.00329 |
| strength | Texture NGTDM 3D | 0.00327 |

**Supplemental Table 7. Feature importances of the most important features of the Patient level MTV model (model 4)**

| **Feature** | **Feature group** | **Feature importance** |
| --- | --- | --- |
| inverse difference normalised | Texture GLCM 3D | 0.00747 |
| minor axis length | Morphology | 0.00712 |
| center of mass shift | Morphology | 0.00711 |
| volume at int fraction 90 | intensity volume | 0.00701 |
| busyness | Texture NGTDM 3D | 0.00673 |
| sphericity | Morphology | 0.00666 |
| strength | Texture NGTDM 3D | 0.00665 |
| Minimum histogram gradient | Intensity histogram | 0.00653 |
| Compactness 1 | Morphology | 0.00643 |
| maximum 3D diameter | Morphology | 0.00638 |
| Spherical disproportion | Morphology | 0.00622 |
| Compactness 2 | Morphology | 0.00618 |
| inverse difference normalised | Texture GLCM 3D | 0.00586 |
| High dependence high grey level emphasis | Texture NGLDM 3D | 0.00567 |
| Grey level non uniformity | Texture GLRLM 3D | 0.00567 |
| strength | Texture NGTDM 2D | 0.00561 |
| Grey level non uniformity | Texture GLRLM 2D | 0.00560 |
| Grey level non uniformity | Texture GLRLM 3D | 0.00546 |
| Grey level non uniformity | Texture GLDZM 2D | 0.00518 |
| Grey level non uniformity | Texture NGLDM 2D | 0.00499 |
| inverse difference normalised | Texture GLCM 2D | 0.00490 |
| least axis length | Morphology | 0.00484 |
| inverse difference moment normalised | Texture GLCM 3D | 0.00476 |
| Grey level non uniformity | Texture NGLDM 3D | 0.00468 |
| inverse difference moment normalised | Texture GLCM 2D | 0.00451 |
| inverse difference normalised | Texture GLCM 2D | 0.00449 |
| Zone distance non uniformity | Texture GLDZM 2D | 0.00442 |
| inverse difference moment normalised | Texture GLCM 3D | 0.00440 |
| Grey level non uniformity | Texture GLRLM 2D | 0.00440 |
| busyness | Texture NGTDM 2D | 0.00439 |
| Grey level non uniformity | Texture GLDZM 3D | 0.00438 |
| Grey level non uniformity | Texture GLSZM 2D | 0.00434 |
| Grey level variance | Texture GLDZM 2D | 0.00430 |
| vol density AEE | Morphology | 0.00429 |
| area density AABB | Morphology | 0.00428 |
| coarseness | Texture NGTDM 2D | 0.00428 |
| minimum | Statistics | 0.00425 |
| difference vol at int fraction | intensity volume | 0.00424 |
| correlation | Texture GLCM 3D | 0.00421 |
| Low dependence low grey level emphasis | Texture NGLDM 3D | 0.00420 |
| contrast | Texture NGTDM 2D | 0.00419 |
| Surface | Morphology | 0.00414 |
| Zone distance non uniformity | Texture GLDZM 3D | 0.00413 |
| Large zone high grey level emphasis | Texture GLSZM 2D | 0.00405 |
| coarseness | Texture NGTDM 3D | 0.00402 |
| inverse difference moment normalised | Texture GLCM 2D | 0.00398 |
| Grey level non uniformity | Texture GLSZM 3D | 0.00397 |
| contrast | Texture NGTDM 3D | 0.00396 |
| major axis length | Morphology | 0.00395 |
| elongation | Morphology | 0.00394 |

**Supplemental Table 8. Feature importances of the most important features of the Maximum model (model 5)**

| **Feature** | **Feature group** | **Feature importance** |
| --- | --- | --- |
| High dependence high grey level emphasis | Texture NGLDM 3D | 0.01280 |
| Large zone high grey level emphasis | Texture GLSZM 2D | 0.00894 |
| vol density AEE | Morphology | 0.00777 |
| High dependence high grey level emphasis | Texture NGLDM 2D | 0.00754 |
| busyness | Texture NGTDM 3D | 0.00748 |
| Small distance low grey level emphasis | Texture GLDZM 3D | 0.00617 |
| Large zone high grey level emphasis | Texture GLSZM 2D | 0.00565 |
| inverse difference moment normalised | Texture GLCM 3D | 0.00563 |
| Grey level non uniformity | Texture GLRLM 2D | 0.00551 |
| minimum | Statistics | 0.00521 |
| Grey level non uniformity | Texture GLSZM 2D | 0.00512 |
| Small zone low grey level emphasis | Texture GLSZM 2D | 0.00510 |
| Grey level non uniformity | Texture NGLDM 2D | 0.00508 |
| busyness | Texture NGTDM 2D | 0.00504 |
| first measure of information correlation | Texture GLCM 2D | 0.00491 |
| inverse difference moment normalised | Texture GLCM 2D | 0.00488 |
| first measure of information correlation | Texture GLCM 3D | 0.00485 |
| High dependence high grey level emphasis | Texture NGLDM 2D | 0.00478 |
| asphericity | Morphology | 0.00475 |
| Grey level non uniformity | Texture GLRLM 2D | 0.00471 |
| Small zone low grey level emphasis | Texture GLSZM 3D | 0.00463 |
| High dependence low grey level emphasis | Texture NGLDM 3D | 0.00452 |
| inverse difference moment normalised | Texture GLCM 3D | 0.00446 |
| Large zone low grey level emphasis | Texture GLSZM 2D | 0.00441 |
| first measure of information correlation | Texture GLCM 2D | 0.00440 |
| Grey level non uniformity | Texture GLRLM 3D | 0.00439 |
| Low grey level zone emphasis | Texture GLSZM 3D | 0.00434 |
| Large zone emphasis | Texture GLSZM 3D | 0.00423 |
| Large zone high grey level emphasis | Texture GLSZM 3D | 0.00422 |
| Morans I | Morphology | 0.00421 |
| Grey level non uniformity | Texture GLRLM 2D | 0.00415 |
| inverse difference normalised | Texture GLCM 3D | 0.00414 |
| minor axis length | Morphology | 0.00413 |
| inverse difference normalised | Texture GLCM 3D | 0.00412 |
| center of mass shift | Morphology | 0.00400 |
| Large distance low grey level emphasis | Texture GLDZM 2D | 0.00400 |
| Grey level non uniformity | Texture NGLDM 2D | 0.00396 |
| Volume | Morphology | 0.00395 |
| maximum 3D diameter | Morphology | 0.00395 |
| Grey level non uniformity | Texture GLRLM 3D | 0.00394 |
| Grey level non uniformity | Texture GLDZM 2D | 0.00393 |
| Large zone low grey level emphasis | Texture GLSZM 2D | 0.00390 |
| Surface | Morphology | 0.00388 |
| elongation | Morphology | 0.00383 |
| Short run low grey level emphasis | Texture GLRLM 2D | 0.00379 |
| correlation | Texture GLCM 3D | 0.00378 |
| Long run low grey level emphasis | Texture GLRLM 2D | 0.00373 |
| volume at int fraction 90 | intensity volume | 0.00370 |
| Large zone emphasis | Texture GLSZM 2D | 0.00370 |
| skewness | Intensity histogram | 0.00369 |

**Supplemental Table 9. Feature importances of the most important features of the Median model (model 6)**

| **Feature** | **Feature group** | **Feature importance** |
| --- | --- | --- |
| volume at intensity fraction 10 | intensity volume | 0.00720 |
| difference volume at intensity fraction | intensity volume | 0.00658 |
| major axis length | Morphology | 0.00591 |
| Short run low grey level emphasis | Texture GLRLM 2D | 0.00577 |
| flatness | Morphology | 0.00560 |
| minimum | Statistics | 0.00557 |
| inverse difference moment normalised | Texture GLCM 2D | 0.00526 |
| Grey level variance GLDZM | Texture GLDZM 2D | 0.00499 |
| vol density AEE | Morphology | 0.00493 |
| skewness | Statistics | 0.00492 |
| Compactness 1 | Morphology | 0.00482 |
| Small distance low grey level emphasis | Texture GLDZM 3D | 0.00481 |
| asphericity | Morphology | 0.00471 |
| Short run low grey level emphasis | Texture GLRLM 2D | 0.00465 |
| sphericity | Morphology | 0.00459 |
| first measure of information correlation | Texture GLCM 2D | 0.00452 |
| Morans I | Morphology | 0.00441 |
| kurtosis | Statistics | 0.00440 |
| vol density AABB | Morphology | 0.00438 |
| Long run high grey level emphasis | Texture GLRLM 2D | 0.00436 |
| first measure of information correlation | Texture GLCM 2D | 0.00435 |
| Spherical disproportion | Morphology | 0.00431 |
| Compactness 2 | Morphology | 0.00430 |
| skewness | Intensity histogram | 0.00425 |
| coarseness | Texture NGTDM 3D | 0.00424 |
| Grey level non uniformity | Texture GLSZM 2D | 0.00416 |
| int at vol fraction 90 | intensity volume | 0.00406 |
| mode | Intensity histogram | 0.00404 |
| elongation | Morphology | 0.00400 |
| 10th percentile | Statistics | 0.00391 |
| Long run high grey level emphasis | Texture GLRLM 2D | 0.00389 |
| minor axis length | Morphology | 0.00385 |
| center of mass shift | Morphology | 0.00384 |
| mean | First order | 0.00384 |
| angular second moment | Texture GLCM 2D | 0.00383 |
| Zone size non uniformity | Texture GLSZM 3D | 0.00383 |
| Long run high grey level emphasis | Texture GLRLM 3D | 0.00379 |
| joint maximum | Texture GLCM 2D | 0.00372 |
| Small zone low grey level emphasis | Texture GLSZM 3D | 0.00368 |
| Small zone low grey level emphasis | Texture GLSZM 2D | 0.00368 |
| maximum 3D diameter | Morphology | 0.00365 |
| Grey level non uniformity normalized | Texture GLRLM 2D | 0.00360 |
| busyness | Texture NGTDM 2D | 0.00357 |
| Surface | Morphology | 0.00354 |
| first measure of information correlation | Texture GLCM 2D | 0.00353 |
| kurtosis | Intensity histogram | 0.00351 |
| joint maximum | Texture GLCM 2D | 0.00350 |
| volume at int fraction 90 | intensity volume | 0.00343 |
| first measure of information correlation | Texture GLCM 2D | 0.00340 |
| approximate volume | Morphology | 0.00337 |

**Supplemental Table 10. Feature importances of the most important features of the Dissemination model (model 7)**

| **Feature** | **Feature group** | **Feature importance** |
| --- | --- | --- |
| DmaxBulk | Dissemination | 0.06084 |
| SpreadBulk | Dissemination | 0.05621 |
| DmaxPatient | Dissemination | 0.04942 |
| Original mean | First order | 0.04934 |
| VolSpreadBulk | Dissemination | 0.04823 |
| VolSpreadPatient | Dissemination | 0.04791 |
| ExactVolume | First order | 0.04713 |
| DvolPatient | Dissemination | 0.04599 |
| Original TLG | First order | 0.04493 |
| SpreadPatient | Dissemination | 0.04492 |
| DSUVmaxSumBulk | Dissemination | 0.04292 |
| DSUVmaxSumHot | Dissemination | 0.04029 |
| DSUVpeakSumPatient | Dissemination | 0.03931 |
| DSUVmaxSumPatient | Dissemination | 0.03810 |
| DSUVpeakSumHot | Dissemination | 0.03795 |
| Original max | First order | 0.03689 |
| local intensity peak | First order | 0.03680 |
| global intensity peak | First order | 0.03521 |
| DSUVpeakSumBulk | Dissemination | 0.03435 |
| DSUVmaxBulk | Dissemination | 0.03393 |
| DSUVpeakBulk | Dissemination | 0.03330 |
| NumberLesions | Dissemination | 0.03252 |
| DSUVmaxPatient | Dissemination | 0.03251 |
| DSUVpeakPatient | Dissemination | 0.03099 |

**Supplemental Table 11. Feature importances of the most important features of the ‘Dissemination + patient level MTV’ model (model 8)**

| **Feature** | **Feature group** | **Feature importance** |
| --- | --- | --- |
| DmaxBulk | Dissemination | 0.01000 |
| SpreadBulk | Dissemination | 0.00897 |
| DmaxPatient | Dissemination | 0.00747 |
| volume at int fraction 90 | intensity volume | 0.00739 |
| strength | Texture NGTDM 3D | 0.00739 |
| busyness | Texture NGTDM 3D | 0.00632 |
| Compactness 2 | Morphology | 0.00630 |
| strength | Texture NGTDM 2D | 0.00590 |
| maximum 3D diameter | Morphology | 0.00588 |
| Compactness 1 | Morphology | 0.00587 |
| inverse difference normalised | Texture GLCM 3D | 0.00568 |
| inverse difference normalised | Texture GLCM 3D | 0.00562 |
| inverse difference normalised | Texture GLCM 2D | 0.00550 |
| VolSpreadPatient | Dissemination | 0.00547 |
| Grey level non uniformity | Texture NGLDM 3D | 0.00539 |
| sphericity | Morphology | 0.00536 |
| minor axis length | Morphology | 0.00534 |
| coarseness | Texture NGTDM 3D | 0.00528 |
| Spherical disproportion | Morphology | 0.00525 |
| volume at int fraction 10 | intensity volume | 0.00512 |
| Grey level non uniformity | Texture GLRLM 3D | 0.00497 |
| center of mass shift | Morphology | 0.00489 |
| inverse difference moment normalised | Texture GLCM 3D | 0.00476 |
| Minimum histogram gradient | Intensity histogram | 0.00473 |
| Grey level non uniformity | Texture NGLDM 2D | 0.00468 |
| Grey level non uniformity | Texture GLRLM 2D | 0.00467 |
| least axis length | Morphology | 0.00465 |
| busyness | Texture NGTDM 2D | 0.00463 |
| SpreadPatient | Dissemination | 0.00450 |
| Grey level non uniformity | Texture GLSZM 2D | 0.00447 |
| inverse difference moment normalised | Texture GLCM 3D | 0.00431 |
| High dependence high grey level emphasis | Texture NGLDM 3D | 0.00430 |
| vol density AEE | Morphology | 0.00422 |
| Grey level non uniformity | Texture GLRLM 2D | 0.00409 |
| inverse difference normalised | Texture GLCM 2D | 0.00404 |
| Zone distance non uniformity | Texture GLDZM 2D | 0.00400 |
| VolSpreadBulk | Dissemination | 0.00396 |
| difference vol at int fraction | intensity volume | 0.00396 |
| skewness | Statistics | 0.00396 |
| NumberLesions | Dissemination | 0.00395 |
| area density AABB | Morphology | 0.00395 |
| Grey level non uniformity | Texture GLRLM 3D | 0.00395 |
| inverse difference moment normalised | Texture GLCM 2D | 0.00391 |
| first measure of information correlation | Texture GLCM 2D | 0.00383 |
| Grey level non uniformity | Texture GLSZM 3D | 0.00382 |
| kurtosis | Intensity histogram | 0.00379 |
| elongation | Morphology | 0.00372 |
| Grey level variance | Texture GLDZM 2D | 0.00372 |
| inverse difference moment normalised | Texture GLCM 2D | 0.00371 |
| major axis length | Morphology | 0.00370 |

**Supplemental Table 12. Feature importances of the most important features of the ‘Dissemination + largest’ model (model 9)**

| **Feature** | **Feature group** | **Feature importance** |
| --- | --- | --- |
| DmaxBulk | Dissemination | 0.01531 |
| DmaxPatient | Dissemination | 0.01136 |
| SpreadBulk | Dissemination | 0.01091 |
| SpreadPatient | Dissemination | 0.00796 |
| VolSpreadPatient | Dissemination | 0.00774 |
| strength 2 | Texture NGTDM 3D | 0.00559 |
| Low dependence low grey level emphasis 2 | Texture NGLDM 3D | 0.00552 |
| minimum | Statistics | 0.00552 |
| NumberLesions | Dissemination | 0.00550 |
| Grey level variance | Texture GLDZM 2D | 0.00546 |
| Grey level non uniformity | Texture NGLDM 2D | 0.00536 |
| DSUVpeakSumPatient | Dissemination | 0.00524 |
| busyness | Texture NGTDM 3D | 0.00508 |
| busyness | Texture NGTDM 2D | 0.00505 |
| Low dependence low grey level emphasis | Texture NGLDM 2D | 0.00501 |
| dependence Count Energy | Texture NGLDM 2D | 0.00492 |
| inverse difference moment normalised | Texture GLCM 3D | 0.00482 |
| Grey level non uniformity | Texture GLSZM 2D | 0.00481 |
| Minimum histogram gradient | Intensity histogram | 0.00474 |
| area density AABB | Morphology | 0.00474 |
| volume at int fraction 90 | intensity volume | 0.00465 |
| DSUVmaxSumPatient | Dissemination | 0.00465 |
| Grey level non uniformity | Texture GLDZM 2D | 0.00454 |
| VolSpreadBulk | Dissemination | 0.00449 |
| inverse difference moment normalised | Texture GLCM 2D | 0.00449 |
| correlation | Texture GLCM 2D | 0.00437 |
| strength | Texture NGTDM 2D | 0.00436 |
| correlation | Texture GLCM 2D | 0.00434 |
| Grey level non uniformity | Texture NGLDM 2D | 0.00429 |
| DSUVpeakSumBulk | Dissemination | 0.00424 |
| Large zone high grey level emphasis | Texture GLSZM 2D | 0.00422 |
| inverse difference normalised | Texture GLCM 3D | 0.00420 |
| Morans I | Morphology | 0.00413 |
| minor axis length | Morphology | 0.00412 |
| Grey level non uniformity | Texture GLRLM 2D | 0.00411 |
| Compactness 1 | Morphology | 0.00406 |
| High dependence high grey level emphasis | Texture NGLDM 2D | 0.00397 |
| inverse difference moment normalised | Texture GLCM 3D | 0.00396 |
| Grey level non uniformity | Texture GLRLM 3D | 0.00388 |
| correlation | Texture GLCM 3D | 0.00384 |
| Large zone emphasis | Texture GLSZM 3D | 0.00382 |
| inverse difference moment normalised | Texture GLCM 2D | 0.00382 |
| elongation | Morphology | 0.00382 |
| Large distance low grey level emphasis | Texture GLDZM 2D | 0.00379 |
| sphericity | Morphology | 0.00370 |
| Spherical disproportion | Morphology | 0.00367 |
| Large distance low grey level emphasis | Texture GLDZM 2D | 0.00367 |
| inverse difference moment normalised | Texture GLCM 2D | 0.00365 |
| High dependence high grey level emphasis | Texture NGLDM 3D | 0.00362 |
| Zone distance non uniformity | Texture GLDZM 2D | 0.00362 |

**Supplemental Table 13. Feature importances of the most important features of the ‘Dissemination + hottest’ model (model 10)**

| **Feature** | **Feature group** | **Feature importance** |
| --- | --- | --- |
| DmaxBulk | Dissemination | 0.01504 |
| SpreadBulk | Dissemination | 0.01311 |
| DmaxPatient | Dissemination | 0.01212 |
| VolSpreadPatient | Dissemination | 0.01198 |
| ExactVolume | First order | 0.01050 |
| SpreadPatient | Dissemination | 0.00948 |
| VolSpreadBulk | Dissemination | 0.00911 |
| DSUVpeakSumPatient | Dissemination | 0.00793 |
| NumberLesions | Dissemination | 0.00787 |
| Original TLG | First order | 0.00759 |
| DSUVmaxSumPatient | Dissemination | 0.00618 |
| DvolPatient | Dissemination | 0.00559 |
| busyness | Texture NGTDM 2D | 0.00481 |
| DSUVmaxSumHot | Dissemination | 0.00477 |
| area density AABB | Morphology | 0.00469 |
| DSUVpeakSumHot | Dissemination | 0.00466 |
| dependence Count Energy | Texture NGLDM 2D | 0.00440 |
| elongation | Morphology | 0.00424 |
| strength | Texture NGTDM 3D | 0.00417 |
| busyness | Texture NGTDM 3D | 0.00411 |
| angular second moment | Texture GLCM 2D | 0.00411 |
| Large distance low grey level emphasis | Texture GLDZM 2D | 0.00410 |
| Large zone low grey level emphasis | Texture GLSZM 2D | 0.00391 |
| flatness | Morphology | 0.00374 |
| Low dependence low grey level emphasis | Texture NGLDM 2D | 0.00369 |
| Morans I | Morphology | 0.00365 |
| Minimum histogram gradient grey level | Intensity histogram | 0.00363 |
| Spherical disproportion | Morphology | 0.00359 |
| first measure of information correlation | Texture GLCM 2D | 0.00358 |
| skewness | Intensity histogram | 0.00351 |
| Grey level variance | Texture GLDZM 2D | 0.00350 |
| joint maximum | Texture GLCM 2D | 0.00350 |
| joint maximum | Texture GLCM 2D | 0.00342 |
| DSUVpeakSumBulk | Dissemination | 0.00341 |
| Grey level non uniformity | Texture GLSZM 2D | 0.00338 |
| inverse difference moment normalised | Texture GLCM 2D | 0.00337 |
| High dependence low grey level emphasis | Texture NGLDM 2D | 0.00331 |
| cluster shade | Texture GLCM 3D | 0.00331 |
| vol density AEE | Morphology | 0.00329 |
| kurtosis | Statistics | 0.00327 |
| Grey level non uniformity | Texture GLRLM 2D | 0.00326 |
| asphericity | Morphology | 0.00324 |
| Compactness | Morphology | 0.00320 |
| volume at int fraction 90 | intensity volume | 0.00319 |
| Maximum histogram gradient grey level | Intensity histogram | 0.00319 |
| Low dependence low grey level emphasis | Texture NGLDM 3D | 0.00319 |
| coarseness | Texture NGTDM 2D | 0.00316 |
| cluster shade 2 | Texture GLCM 2D | 0.00313 |
| Grey level non uniformity normalized | Texture NGLDM 2D | 0.00312 |
| Large zone low grey level emphasis | Texture GLSZM 2D | 0.00311 |
